# Construction of Piano Performance Curriculum System Based on Convolutional Neural Network

**DOI:** 10.1155/2022/1556606

**Published:** 2022-08-23

**Authors:** Dongxu Yang, Weiya Zhang

**Affiliations:** ^1^School of Music, Shandong Women's University, Jinan, Shandong 250003, China; ^2^New Era University College, Kajang, Selangor 43000, Malaysia; ^3^School of Art, Jinan Preschool Education College, Jinan, Shandong 250307, China

## Abstract

Comprehensively promoting quality education and the all-round development of human beings is the focus of current educational work. When carrying out quality education at university, it is important to start from all aspects such as ideology and morality, physical and mental health, professional learning and personality cultivation, and to give full play to their potential and enhance their creativity. Music teaching is an important element of quality education and using it as an entry point can prevent it from being too abstract. However, music education is still a weak aspect of higher education in China.

## 1. Introduction

Music as a multimedia information is becoming more and more informative in its own right, and at the same time more and more demanding for users, which requires an in-depth study of it. Faced with the massive amount of nonexact music information, users have to spend considerable time and effort in order to find their favourite songs. For this challenge, the music recommendation algorithm is proposed and applied [1]. Based on the behavioural characteristics of the user and the characteristics of the music data, it predicts them and pushes them proactively [2]. Its technical route has evolved from good recommendations based on user behaviour at the beginning to association recommendations later and gradually to the mining of potential preferences of users. On the whole, recommendation algorithms have high recommendation performance [3].

However, in the era of big data, recommendation methods based on massive music data also face new challenges and opportunities [4]. On the one hand, the processing of large amounts of data is becoming increasingly complex and requires adjustments to the individual requirements of users, thus improving the original recommendation algorithms. In addition, traditional recommendation algorithms face problems such as “cold starts”, which require further research [5]. On the other hand, new computer technologies such as big data processing technology and deep learning technology are emerging, providing strong support for users to design recommendation algorithms with strong processing power and high accuracy rate [6].

To this end, the author proposes the idea of a “convolutional neural network-based piano performance course module” to enable teachers to better adapt their teaching to the employment needs of students and the comprehensive needs of society for high-quality and high-level talent.

## 2. Introduction to the Theory

### 2.1. The Importance of Piano Teaching

Music movement is very important to the quality of education, so piano movement and music movement are relatively independent disciplines and the most basic disciplines of music movement [7]. So, it is very important to design course content and lecture. Its teaching effect directly affects the core training of Chinese music teachers and plays a key role in improving the overall quality of society.

Using the piano is based on the training of qualified Musiklehrern as the center, which is an important part of the national Musikausbildung piano lessons. The goal is to improve the whole Musikqualitat students [8], so that students understand and master music in class, Musikqualitat strengthens the students for reform. In order to meet the needs of primary school teachers, it is also important to serve the society [9].

### 2.2. Convolutional Neural Networks

A convolutional neural network is a forward network consisting of convolutional operations and a depth structure, and is a typical algorithm for deep learning [10].

Convolutional Berger is widely used in many ways for phantoms, natural speech, and other applications. Convolutional neural networks can be used for classification, retrieval, recognition (classification, regression), segmentation, feature extraction, localisation of key points (pose recognition), etc., [11].

**Figure 1:**
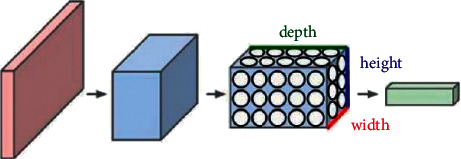
Convolutional neural network schematic diagram.

As shown in Figure 1, a convolutional neural network consists of an input layer, a convolutional layer, an activation function, a pooling layer, and a fully connected layer [11].

The convolutional layer is the core of the neural network, also known as the convolutional kernel, which is divided into size and depth, commonly 3 × 3, 5 × 5, and 11 × 11. The size and depth of the convolutional kernel are determined manually, and the weighting coefficients are initialized using programming methods and optimized to achieve the best classification results. The convolution method uses these weights to perform RGB operations on the image to obtain information about the data in the image [12].

**Figure 2:**
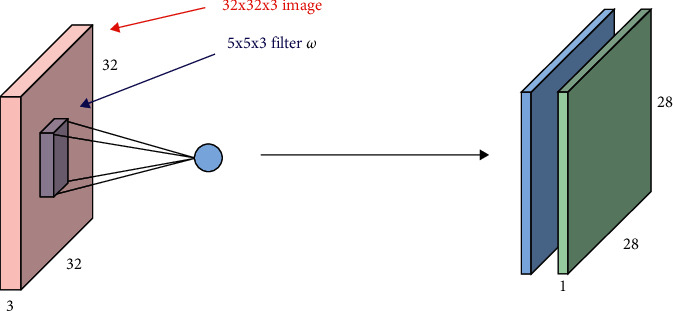
Principle diagram of feature extraction.

The convolution method is not only effective in extracting the image information in the image, but also enables the dimensionality reduction of the image. Its feature extraction schematic is shown in Figure 2. To make the size of the convolved feature values the same as the original image, the padding value (full zero padding) must be set to SAME (if VALID is unpadded), where *i* is the input frame, *k* is the convolution kernel size and strides are the motion steps (moving distance >1 also enables dimensionality reduction) [13].

Through the folded neural network (root), it can be used for classification, searching, recognition (classification and backtracking), Faux-band effect, locating key (pleasure recognition), and other classification. We extracted a value from the given size (equal to the size of the false core) to represent the value by scrolling (false). If folded, the calculated number of Rubik's cubes (all of the same size) can get several kinds of samples[14].

**Figure 3:**
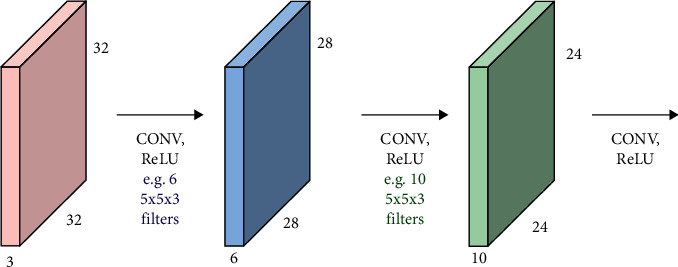
Principle diagram of feature map extraction.

In addition to said input image, the convolutional objects described in Figure 3 may also be said feature maps [15].

## 3. Research Methodology Design

### 3.1. Course System Model

**Figure 4:**
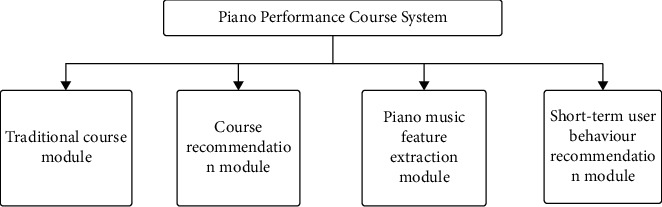
Model of the curriculum system.

Piano electives based on neural folding networks include a traditional course plug-in, a page-bubble plug-in, a plug-in for piano manual separation, and a plug-in to prompt short-term user behaviour. A modular component diagram is shown in Figure 4.

It can be seen from the above figure that the piano performance course module constructed this time is a set of comprehensive and coordinated system, which can recommend corresponding piano lessons and basic courses according to individual ability and historical performance. Among them, recommendation class and piano accompaniment class are to cultivate students' playing skills and the ability to use knowledge, while music work appreciation and piano art history class are aimed at improving students' comprehensive quality, while piano teaching class is aimed at improving students' ability. On this basis, the author hopes to make some contributions to the construction of piano course system for piano performance specialty in China, so as to provide more high-quality course system for basic education in China [16].

### 3.2. CNN Training Model

**Figure 5:**
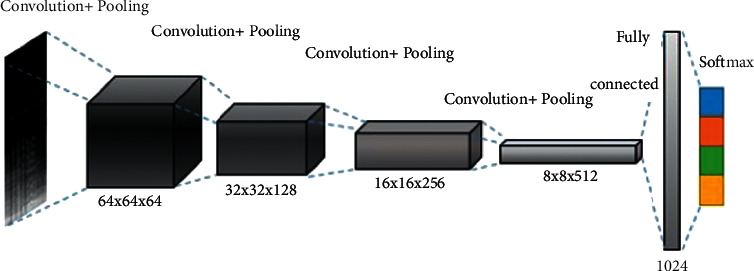
CNN training model.

LeNet 5 is a relatively typical folded network model used in many image recognition programs, including manual writing of numbers and accessory license plates. The training model in this paper is based on LeNet 5, and other outstanding CNN models [17] are also used, which have been modified several times during the experiment. The final model is shown in Figure 5.

### 3.3. Piano Performance Course Recommendations

In this paper, CNN classification mode is used to reserve a basic attribute dimension for the classification of music in piano class [18]. Optimizing units can produce more subtle units. These libraries can be used to measure the similarity of music and calculate the characteristics of users [19]. If the music library already contains the user's favourite music, then the visit signature library can be queried directly. If you do not use the CNN model, you have to use the model there to predict and classify features. Based on these classical characteristics, this paper proposes the following recommended processes [20].

A CNN classification model is trained, whose CNN is a method that can predict, classify and categorise. User features are calculated by analysing the interrelationship between piano music and category features and the relationship with the user to determine the relationship between user and category features [21].

## 4. Implementation and Analysis of Results

### 4.1. Implementation of Piano Music Classification Based on Convolutional Neural Network

#### 4.1.1. CNN Training Process and Experimental Environment

Its basic working process can be divided into three stages: music files are divided into two categories: a training category and a detection category. Secondly, their sounds are segmented, and then a map of spectral characteristics is generated. The spectral characteristics of the samples are then compressed and introduced into the neural network for training so as to obtain their weighting. Experimental data are classified using the trained neural network and recommendations are made.

#### 4.1.2. Training Data and Test Data

The training case is presented by NetEase and Global Piano Music and is divided into four categories: blues, classical, jazz, and pop, with 100 main notes, all pure piano notes, in mp3 format horizontal axis. The segmented spectrum clip has 128 × 128 pixels and represents a 2.56 s audio signal. For a 4-minute audio data, 93 spectrum segments can be obtained. The segmentation of the audio data for each of the four classifications resulted in approximately 8000 image samples. Forty percent of the image samples were used as training samples, 30 percent as calibration samples, and 30 percent as test samples.

**Figure 6:**
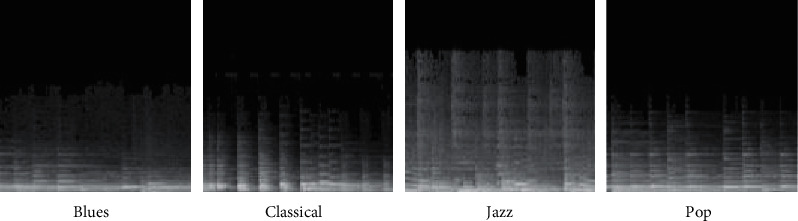
Spectrum samples of the four types of music.

The distribution of the image shows the gray distribution, the *X* axis is the time axis, the *Y* axis is the frequency, the gray level is the frequency, and the white range (the longer gray level) is the larger wavelength.  Figure 6 shows a more stereotypical spectrum used by four different people. Each chart represented a piece of music and concluded that blues generally had a lower margin, with smaller exclusions, indicating that the music was more comfortable. The classical part has higher frequency and reduced amplitude, indicating that the chord with higher pitch is weaker. The constant change and repetition of jazz repertoire results in a higher speed, lower overall pop frequency, and a balanced intensity and strong rhythm.

**Figure 7:**
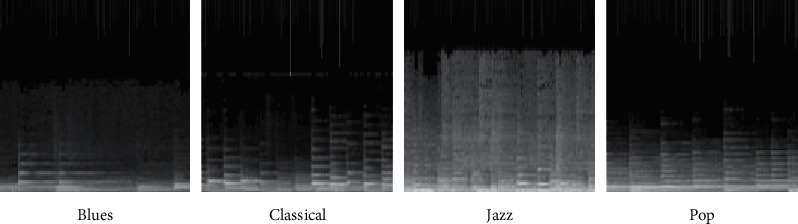
Four types of music spectrum samples combining note features.

To improve the classification accuracy of the CNN, a number of note characteristics were added to the original spectrum. The 128 note fundamental frequencies were used as pitch characteristics before playing each piano piece, from top to bottom, left to right, in the upper part of the spectrum image. In the vertical direction the note fundamental frequencies are compressed vertically into 128 levels, with the same 256 levels of gray scale, and the image is shown in Figure 7.

#### 4.1.3. Comparison of Classification Results

*(1) Activation Function Versus Gradient Descent Method*. The result of a fragment muster promise is that the dimensions contain a number of Skalenwerte parts per Skalenwert percentage, including the corresponding category while using Abstimmungsmethode to handle the weight of each component and finally determine each category of components.

**Figure 8:**
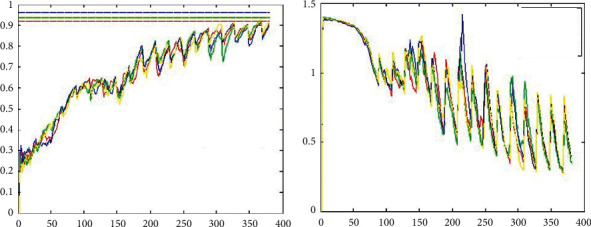
Comparison of four experiments using the ELU activation function (learning rate = 0.001).

As the learning of the neural network is stochastic, the classification results will change each time, therefore, four experiments were conducted with guaranteed learning rate, activation function, and optimal control, and the results are shown in Figure 8.

**Figure 9:**
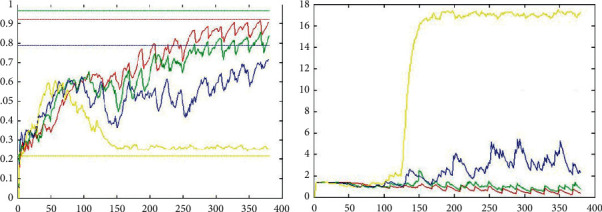
Experimental comparison of 4 learning rates under ELU conditions.

The paper then tests the training of the ELU activation function at various learning rates, as shown in Figure 9. The comparison shows that the ELU is particularly sensitive to the learning rate, with the entire neural network “dying” at rates above 0.003. In terms of accuracy and gradient variation, the optimal learning rate should be between 0.001 and 0.002.

The next paper tested the ELU and the training status of the activation function at different learning rates is shown in Figure 9. The comparison shows that the ELU has a particularly large effect on the learning rate, with the whole neural network dying at 0.003. The optimal learning rate, in terms of both accuracy and gradient variability, should be 0.001–0.002.

**Figure 10:**
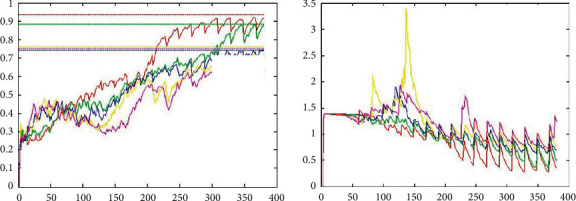
Experimental comparison of the 4 learning rates under ReLU conditions.

The experimental comparison of the four learning rates under the ReLU condition is shown in Figure 10. The rate of gradient change is essentially the same and the number of iterations required to converge is essentially the same, the only difference being that a larger learning rate leads to a larger gradient change, but this is not the best choice. At the same time, the optimal learning speed remains close to 0.001.

**Figure 11:**
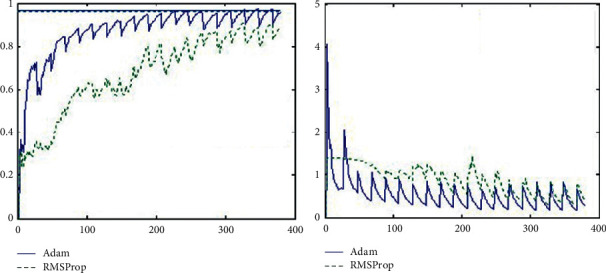
Experimental comparison of Adam and RMSProp.

In addition, the two best controllers, RMSProp and Adam, are compared in this paper and the corresponding experimental results are given. The experimental comparison of Adam and RMSProp is shown in Figure 11. You can see that Adam's gradient descent is better than RMSProp's and the rate of descent is fast. After 50 sharp shocks, Adam quickly finds the right direction and stabilises very quickly.

The results show that with ELU, Adam and a learning rate of 0.001, the classification accuracy of the CNN is higher than 0.96. Therefore, the above two learning methods are chosen in this paper because the correct classification results will be of great help to the subsequent recommendations.

### 4.2. Realisation of the Traditional Curriculum

#### 4.2.1. Piano Accompaniment Courses

The piano accompaniment is a special instrument, which is a new kind of music creation, it plays a pivotal role in instrumental, vocal, and dance performances, and its perfect cooperation with other actors can fully demonstrate the charm of the music and bring a visual enjoyment to the listener.

Piano accompaniment is divided into musical accompaniment. An orthogonal accompaniment is an accompanying piece of music made by the composer when creating a solo piece of music. In formal competitions, performances, and other occasions, most Chinese and foreign classical music is accompanied by a formal score. The piano accompaniment, on the other hand, is mostly used for simple accompaniment, transposed and harmonised, unaccompanied songs, instrumental music, etc. The piano accompaniment class is becoming more and more important. The level of piano accompaniment is directly related to the teacher's ability to do the basic work. The proper accompaniment of the piano with improvisation both stimulates the imagination of the students and combines piano playing skills with comprehensive piano theory. For this reason, the author proposes that piano accompaniment should be made a compulsory subject in university piano teaching.

#### 4.2.2. Music Appreciation Course

This article argues that a music appreciation course is a very important course which can improve students' musical cultivation as well as broaden their horizons, improve their musical composition and raise their level of musical creativity.

The teaching objective of vocal music in higher education is to provide high-quality music teachers for basic education, and it is of great importance to provide them with an all-round and comprehensive study. At present, China's university music majors follow the teaching methods of professional music colleges, focusing only on the teaching of “main professional courses”, resulting in the increasingly prominent phenomenon of “bias” and the lack of comprehensive music studies. For example, teachers and students generally believe that piano and voice are the main subjects and the rest are secondary, so that after graduation, they have mastered very little of piano, voice, symphonic music, chamber music, and folk music, which seriously violates the “comprehensive” teaching purpose of the music education major and makes students incompetent for their future work.

### 4.3. Course Recommendation Module Implementation

In this paper, recommendations are made to individual users based on their behavioural characteristics, and songs with high similarity are recommended to the users. The data used in this experiment are 1000 piano pieces and 100 songs per user. After the classification prediction by CNN, 1000 classification features are derived and optimized. The critical pattern = 0.5, i.e., 0.75, and music above the critical point was considered to have multiple classification features. A music classification model based on HMM (Hidden Markov model) was built to obtain the user's song list in order to more closely match the user's behavioural habits. The HMM classification state transfer probabilities are shown in Table 1, and the recommendation results of the comprehensive evaluation of user features are shown in Table 2.

Using this method can combine the recommendation method of classification features with other methods, so as to achieve the purpose of improving the recognition accuracy. The classification features of CNN only contain the characteristics of music, it can constitute more accurate music characteristics through the title, description, and other textual characteristics. The user's behavioural selection should include the user's personal information, search history, and collection list.

### 4.4. Piano Music Feature Extraction Module Implementation

This equipment use the SoX to create a multidimensional spectrum SoX to full name sun pronunciation change is a very famous and well-known abroad through open source audio processing software. It is also a kind of beyond the resources of the audio format conversion tool because it is in the widely used on audio processing. It has now been transformed into multiple operating system platforms, and compatible with each other.

**Figure 12:**
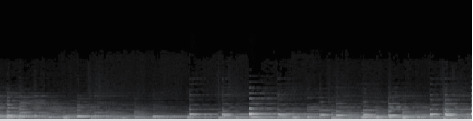
Audio part of the spectrum.

In this experiment, SoX is used to generate the tools for the command line of the music spectrum. The software enables automatic segmentation and spectrum plotting of large amounts of audio data using the “spectrogram” command. The spectrum of the audio part is shown in Figure 12. The spectrogram is displayed as a PNG portable network image with the time on the *X*-axis, the amplitude of the sound signal on the *Y*-axis and the amplitude on the *Z*-axis. The *Z*-axis values are represented in the *XY* plane by the pixel colour (or optionally by the brightness). When an audio signal includes more than one channel, i.e., the left channel of the sound signal, these channels are displayed starting from top to bottom. In this way, a two-dimensional gray-scale image gives a good representation of the multidimensional nature of the sound.

**Figure 13:**
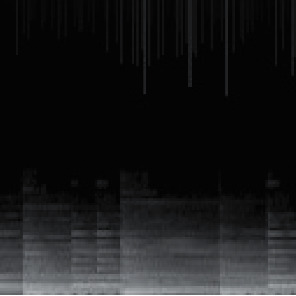
Sample spectrum of notes with superimposed note features.

With the spectrum divided into 128 × 128, the note spectrum samples are superimposed on the upper part of the spectrum, sampling the characteristics of the notes. One of the note spectrum samples of the superimposed note features is shown in Figure 13. In particular, before each performance, a base frequency of 128 tones is drawn from top to bottom and from left to right. In the vertical direction, the pitch fundamental frequencies are compressed to 128 levels, with 256 levels of gray scale.

## 5. Conclusion

In the framework of primary education reform, it is an urgent task to train high-quality new people. Music education is an indispensable part of quality education. This kind of education can promote the harmonious development of body and mind, and also improve the overall quality of people. In this work, we trained CNN network and combined its spectral characteristics with clay characteristics to maintain the characteristics of CNN in the United States. Finally, according to the user's behaviour characteristics and user evaluation, we choose the appropriate course for ourselves, so that everyone can have their own course.

## Figures and Tables

**Table 1:** HMM classification state transfer probability.

	Pop	Jazz	Classical	Blue
Pop	0.775	0.025	0	0.200
Jazz	0.050	0.833	0.067	0.050
Classical	0	0.033	0.833	0.133
Blue	0.406	0	0	0.594

**Table 2:** Recommended results of comprehensive evaluation of user characteristics.

USER	,	Accuracy (%)
Y1	{0.077, 0.049, 0.086, 0.788}	63.5
Y2	{0.056, 0.282, 0.098, 0.563}	17.9
Y3	{0.788, 0.077, 0.054, 0.082}	62.1
Y4	{0.088, 0.805, 0.060, 0.047}	56.8
Y5	{0.036, 0.089, 0.798, 0.077}	45.8
Y6	{0.074, 0.048, 0.072, 0.806}	54.3
Y7	{0.839, 0.068, 0.038, 0.055}	59.2
Y8	{0.271, 0.148, 0.488, 0.092}	28.5
Y9	{0.105, 0.747, 0.097, 0.052}	53.0
Y10	{0.045, 0.097, 0.773, 0.085}	62.4
	average hit rate	50.35

*Note* the average characteristics of users, correct rate is the average click rate of 100 recommendations, and bolded users are multicategory users.

## Data Availability

The dataset used in this paper are available from the corresponding author upon request.
